# Prevalence and incidence of stroke in Latin America and the Caribbean: a systematic review and meta-analysis

**DOI:** 10.1038/s41598-023-33182-3

**Published:** 2023-04-26

**Authors:** Diego Cagna-Castillo, A. Lucia Salcedo-Carrillo, Rodrigo M. Carrillo-Larco, Antonio Bernabé-Ortiz

**Affiliations:** 1 Universidad Cientifica del Sur, Lima, Peru; 2 CRONICAS Center of Excellence in Chronic Diseases, Universidad Peruana Cayetano Heredia, Lima, Peru; 3 Department of Epidemiology and Biostatistics, School of Public Health, Imperial College London, London, UK

**Keywords:** Neurological disorders, Epidemiology, Population screening

## Abstract

Stroke is a recurrent and well-known cardiovascular event and a leading cause of death worldwide. We identified reliable epidemiological evidence of stroke in Latin America and the Caribbean (LAC) and estimated the prevalence and incidence of stroke, overall and by sex, in that region. A systematic search in OVID (Medline, Embase and Global Health) and in the Latin America and Caribbean Health Sciences Literature (LILACS) until the end of 2020 was made for all cross-sectional or longitudinal studies estimating (or allowing the estimation of) the prevalence or incidence of stroke among individuals of the general population ≥ 18 years from LAC countries. No language restriction was applied. Studies were assessed for methodological quality and risk of bias. Pooled estimates were calculated using random effect meta-analysis as high heterogeneity was expected. A total of 31 papers for prevalence and 11 papers for incidence were included in the review for analysis. The overall pooled stroke prevalence was 32 (95% CI 26–38) per 1000 subjects and were similar among men (21; 95% CI 17–25) and women (20; 95% CI 16–23) per 1000 subjects. The overall pooled stroke incidence was 255 (95% CI 217–293) per 100 000 person-years, being higher in men (261; 95% CI 221–301) compared to women (217; 95% CI 184–250) per 100 000 person-years. Our results highlight the relevance of the prevalence and incidence of stroke in the LAC region. The estimates were similar in stroke prevalence by sex, but with higher incidence rates among males than females. Subgroup analyses highlight the need for standardized methodologies to obtain appropriate prevalence and incidence estimates at the population level in a region with a great burden of cardiovascular events.

## Introduction

The World Health Organization (WHO) defines stroke as a “focal or general neurological condition of sudden onset, lasting more than 24 hours or causing death, with no apparent cause apart from being of vascular origin”^1^. Stroke is considered a medical emergency because of the high risk of death with a 28%, 41% and 60% of cumulative risk of death at 28 days, 1 year and 5 years of follow-up, respectively^2^. According to a recent systematic analysis using the Global Burden of Diseases, Injuries, and Risk Factors Study 2019 (GBD) the absolute number of incident and prevalent stroke cases has increased 70% and 85%, respectively, being the second leading cause of death (11.6% of total deaths)^3^. Most of the global burden of stroke is currently focused in low- and middle-income countries (LMIC), which make up more than 65% of the countries in Latin America and the Caribbean (LAC)^4^. While data obtained by the GBD is useful, information from specific regions is also needed to increase our understanding of this condition by comparing epidemiology in different countries and observing changes in incidence in a particular place^5^.

Updated information on stroke, including data on its prevalence and incidence, is crucial for evidence-based stroke care planning and resource allocation. The last systematic review about stroke epidemiology in the LAC region was published in 2003, including only seven studies in South America, and reporting a prevalence ranging from 1.7 to 6.5 per 1,000 subjects, and an incidence ranging between 35 and 183 per 100,000 person-years^6^. It was of note that the prevalence of stroke was relatively low compared to developed countries like the United States in 1991, New Zealand in 1992 and in Italy in 1992 which was 17, 10.2 and 7.3 per 1000 subjects respectively^7–9^; and the findings highlighted that unknown protective ethnic factors, behavioral lifestyles, and eating habits, could explain this difference^6^. According to evidence, at that time in LAC, there was a normocaloric diet based on carbohydrates with long absorption, a high intake of cereals, nuts, and fruits, and a low intake of processed meats, as well as extensive physical activity^10,11^.

However since epidemiological and clinical characteristics of stroke vary according to environmental, racial, sociocultural factors as well as the aging process in LAC, this has led to an increase in cardiovascular risk factors and stroke-related morbidity and mortality rates^12^. It is essential to be aware of the peculiarities of stroke in LAC to reduce the impact of this epidemic. Data such as demographic characteristics; whether they are age, sex and race of the subjects; and epidemiological characteristics; as differences between health systems in the countries, the data collection methodology, the optimal diagnosis and the subtype of stroke. In addition to considering certain limitation of the GBD that are the uncertainties in the measurements, which could generate a possible bias. Limitations such as: not updated census data, missing epidemiological registries in some countries and numerous data sources (surveys, censuses, cohort studies and administrative data) that could lead to inaccuracies in estimating disease burden^13^. Therefore, we aimed to systematize the available evidence to establish estimates of the prevalence and incidence of stroke in the LAC region using population-based studies until the end of 2020.

## Methods

### Data sources and search strategy

A systematic review and meta-analysis (PROSPERO: CRD42021233565) was conducted and reporting was carried out following the Preferred Reporting Items for Systematic Reviews and Meta-Analyses (PRISMA 2020) guidelines^14^. Two main databases were used: OVID (Medline, Embase and Global Health), and the Latin American and Caribbean Health Sciences Literature (LILACS acronym in Spanish). The combination of words used were related to stroke and countries in the LAC region, and the time was defined from database inception until December 31, 2020. The database search was carried out on the 2nd of January 2021. The search terms and strategies employed are shown in Supplementary Table I.

### Study selection and screening

We included full-text studies estimating (or allowing the estimation of) the prevalence and/or incidence of stroke among individuals of the general population ≥ 18 years from the general population (i.e., studies using a random sampling approach to infer results to any specific population, region or country). Individuals enrolled in such these studies must had to have been from LAC countries (i.e., studies conducted with people from LAC in countries abroad, or with foreigners in LAC countries, were not included). Studies could be cross-sectional or longitudinal in nature, and no language restriction was applied. Two authors (DC-C and ALS-C) independently assessed titles and abstracts for eligibility, and disagreements were solved by discussion with a third and fourth reviewer (RMC-L and AB-O). After the initial screening phase, full-text articles were reviewed by the same two authors (DC-C and ALS-C) and disagreements were solved by a third party (AB-O). The agreement between reviewers was evaluated by Cohen’s kappa coefficient (Moderate: 0.41–0.60; Substantial: 0.61–0.80; Almost perfect: 0.81–1.00)^15^.

### Data extraction

Two extraction forms were developed for prevalence and for incidence studies and further tested using a random sample of selected studies. After being tested and approved, these forms were not further changed.

The prevalence template form included the study characteristics such as: publication year, country, scope (subnational or national), year of data collection, study design, age range, mean age, proportion of women, effective sample size, method of diagnosis used (WHO, self-reported, or others), type of prevalence (current, indicating that the subject had a stroke over a specific period of time, or lifetime, indicating that at some point in their life the subject had had a stroke), general estimation of the prevalence of stroke, and prevalence estimates according to sex.

Similarly, the incidence template form included: publication year, country, scope (national or subnational), year of data collection, design (registry or cohort), sample size at baseline, age range at baseline, age mean, proportion of women, method of diagnosis used (WHO, self-reported, or others), type of incidence (cumulative incidence or incidence rate), years of follow-up, stroke estimates of the incidence of stroke estimates, as well as the estimated incidence estimates by sex and among those aged ≥ 35 years and over.

In the case of studies including overall samples (i.e., children, adolescents and adults), if possible, we estimated the prevalence or incidence of those ≥ 18 years (or any other age group over that cutoff, e.g., 25 + or 35 + years). After being tested and approved, these forms were not further changed. Two reviewers (DC-C and ALS-C) extracted the data independently, and disagreements were solved by a third party (RMC-L).

### Assessment of methodological quality

The methodological quality of the included studies was determined using the Critical Appraisal Checklist, which assesses the methodological quality and the possibility of bias in the design, conduction, and analysis of epidemiological studies^16^. The two researchers (DC-C and ALS-C) evaluated the studies included separately, and any disagreement was solved by consensus. Each item was rated as “successfully answered” or “not answered or unclear”. Every “successfully answered” item was valued as 1 point, whereas “not answered or unclear” items were valued as 0. A low risk of bias was defined as ≥ 8 points, and a high risk of bias was determined as < 8 points in the checklist, and this cut-point was used as part included in the sensitivity analysis.

### Data synthesis and analysis

Data analysis was conducted using STATA v16 for Windows (StataCorp, College Station, TX, US). Initially, a brief qualitative description of the prevalence and incidence studies included in the present review was conducted separately.

For meta-analysis of the prevalence of stroke, a pooled estimate (by 1000 subjects) was calculated as a weighted average, by fitting a logistic-normal random-effect model without covariates but with random intercepts. Then, the pooled estimate was calculated using the Freeman-Tukey double arcsine transformation as suggested in the literature^17,18^. Heterogeneity was assessed with the I^2^ statistical test, and because substantial heterogeneity was expected as the studies selected were different in scope (national vs. sub-national), period (current vs. lifetime), and reporting (self-reported vs. other methods), an estimation of random-effects meta-analysis was carried out in specific subgroups. Sensitivity analysis was also performed focusing on age (35 + and 60 + years) and by risk of bias (low vs. high). A meta-analysis stratified by sex was also performed. Finally, meta-regression was also conducted to explain, if possible, between-study heterogeneity.

For the study on the incidence of stroke, a pooled estimate was calculated using incidence rates per 100,000 person-years of follow-up. High heterogeneity similar to the prevalence studies was expected and, therefore, subgroup analyses were carried out according to study characteristics (study type, diagnosis method, scope, and risk of bias). Stratified analyses were also performed, mainly to obtain estimates by sex and age (35 + years). Finally, meta-regression was carried out to explain heterogeneity.

### Ethical approval

Although ethical review was not mandatory because of the use of published data, the protocol of this study work was reviewed and approved by the Institutional Ethics in Investigation Committee of the Universidad Cientifica del Sur (Registration code: 234-2021-PRE15).

## Results

### Selection and inclusion of studies

**Figure 1:**
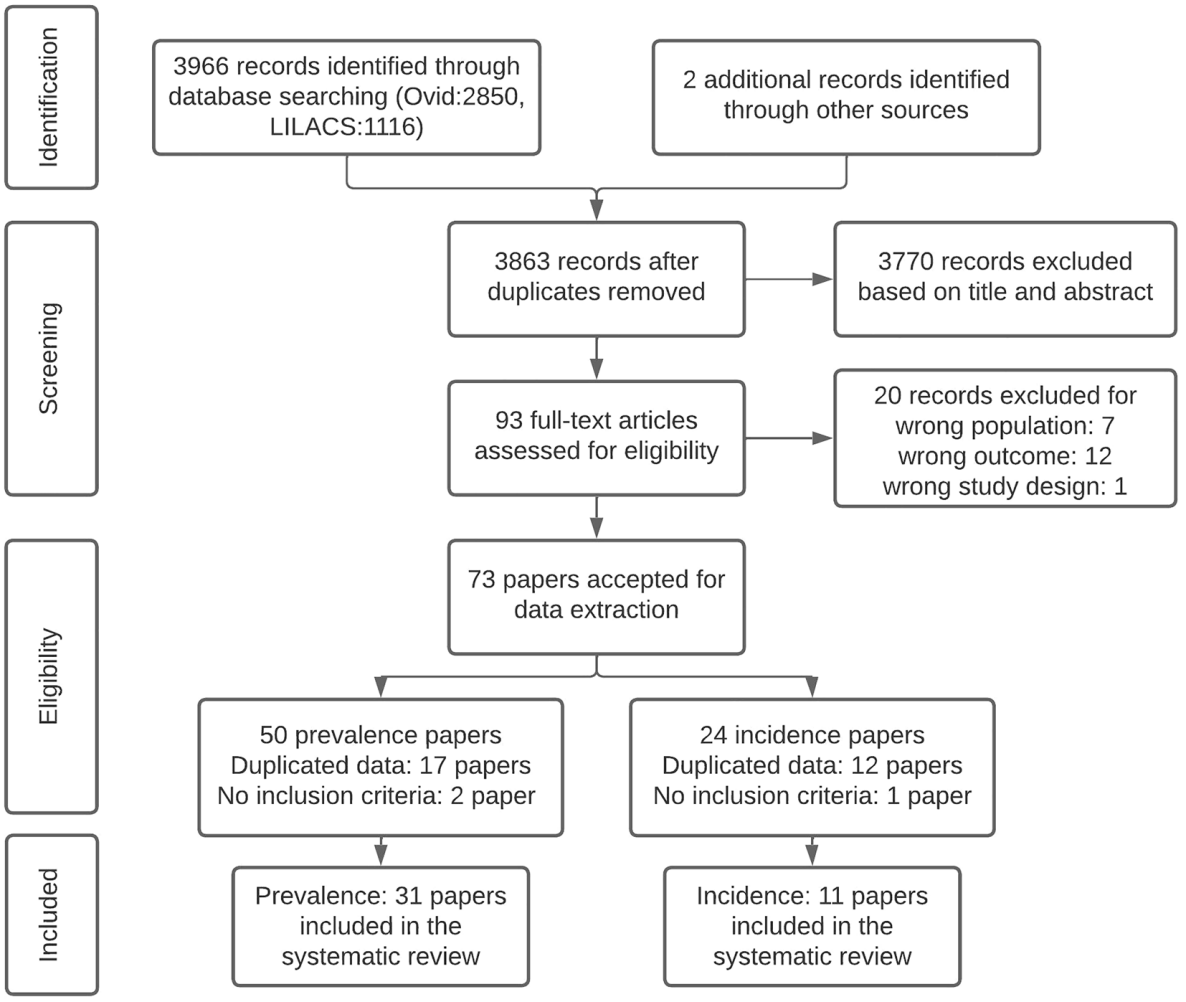
Study selection flowchart. The inclusion and exclusion criteria and the search strategy constructed are shown in detail in the main text and Supplementary Table I.

The initial search strategy identified 3,966 articles and 2 were added from other sources, but only 93 papers were full text assessed for eligibility (Fig. 1). Finally, 31 and 11 papers were included in the prevalence^19–49^ and incidence^33,50–59^ studies, respectively; there was a high agreement between the reviewers with a substantial inter-reliability (kappa value = 0.693). Supplementary Table II.

### Characteristics of selected studies

Manuscripts from 17 countries, published in different languages, were used to estimate the prevalence and incidence of stroke. Of these, 30 were in English, 8 were in Spanish, and 3 were in Portuguese.

**Figure 2:**
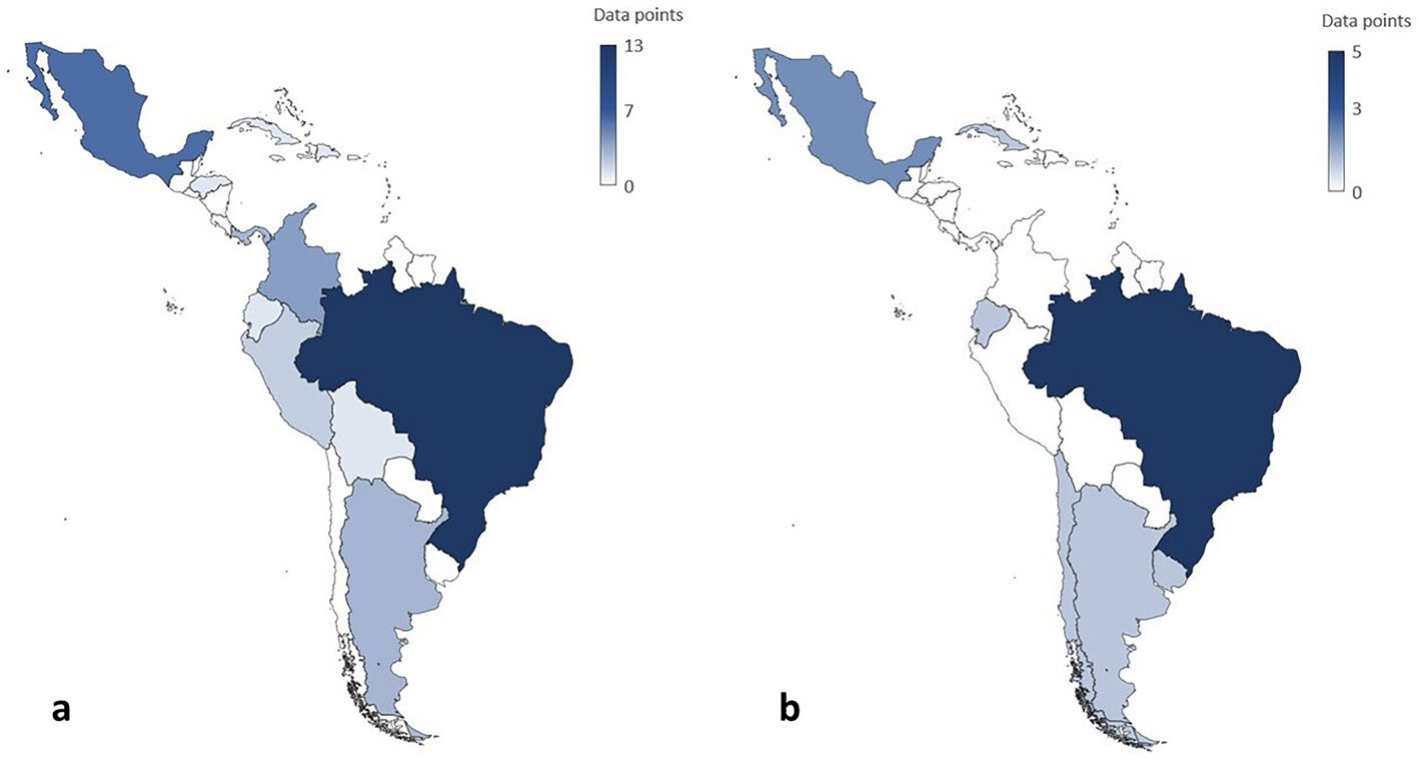
Scientific output map. Total data points for prevalence (**a**) and incidence (**b**) studies in the general population by country and geographic data gaps. Countries in which data were not available are shown in white.

Manuscripts from 13 countries were used to estimate the prevalence of stroke: Brazil contributed with a total of 13 data points, followed by Mexico with 6; Colombia with 4; Argentina and Panama with 3; Peru with 2; and Bolivia, Cuba, the Dominican Republic, Ecuador, Granada, Honduras and Venezuela with 1 data point each (Fig. 2a). Data available for analysis was published from 1988 to 2020. Self-reporting was the most common method used for diagnosis at 64%.

For the estimation of the incidence of stroke, manuscripts from 9 countries were used: Brazil contributed with a total of 5 data points, followed by Mexico and Martinique with 2; and Argentina, Cuba, Ecuador, Barbados, Chile and Uruguay with 1 data point each (Fig. 2b). Data available for analysis was published from 2004 to 2020. The WHO method was most frequently used in 75%.

A qualitative description of the studies included is shown in Supplementary Tables III and IV.

### Quality assessment

**Figure 3:**
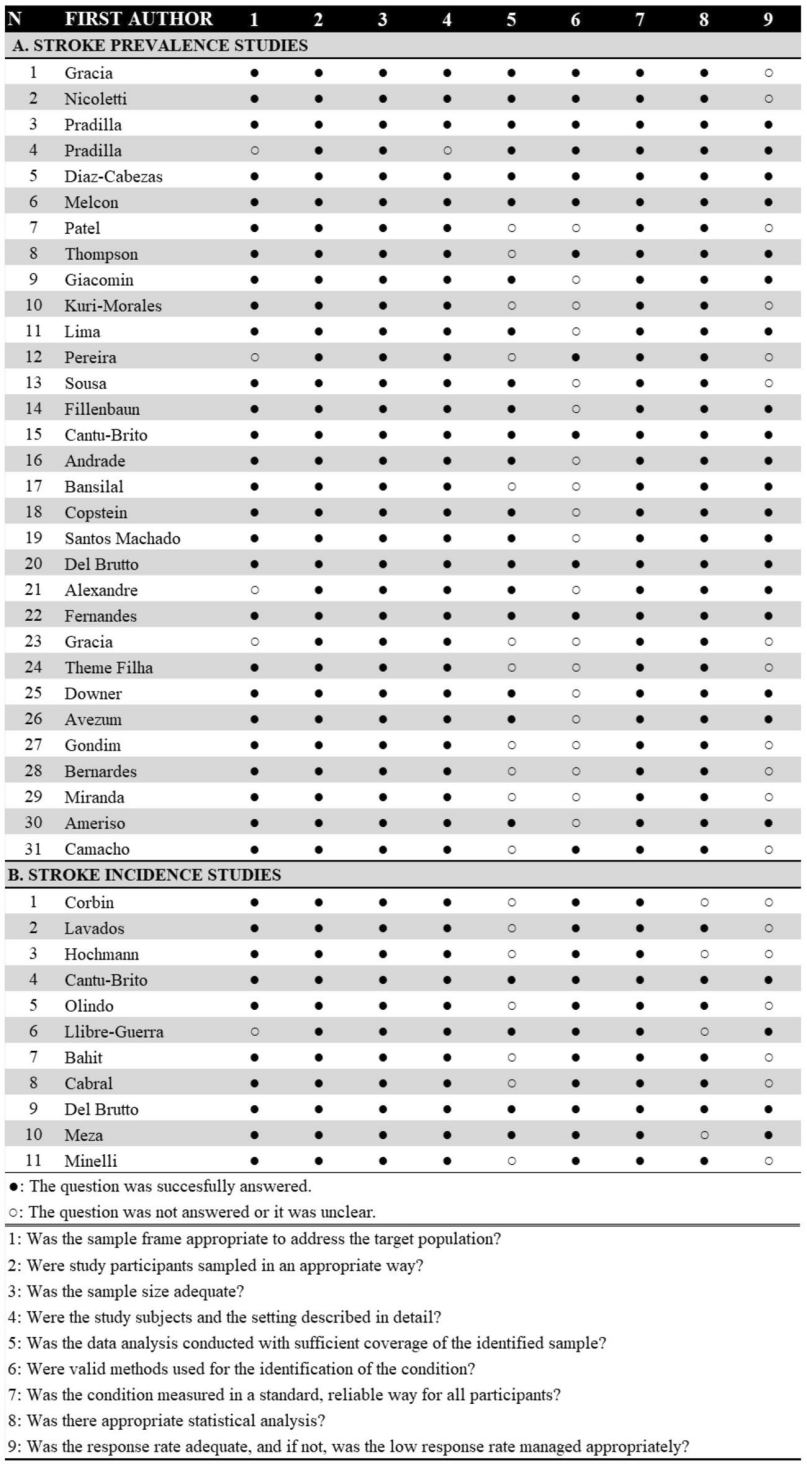
Quality assessment. Assessed with the Critical Appraisal Checklist. (**a**) Prevalence studies. (**b**) Incidence studies. A low risk of bias was defined as ≥ 8 points, and a high risk of bias was defined as < 8 points in the checklist.

Figure 3a shows the quality assessment of the prevalence studies: 8 (25%) studies had high risk of bias and 19 (59%) of the total studies did not mention the use of valid methods for the identification of stroke. Figure 3b shows the results of the quality assessment of the incidence studies: 2 (18%) studies had a high risk of bias.

### Stroke prevalence: pooled estimates

The data available for meta-analysis included 371,260 individuals, with a prevalence ranging from 4 to 87 per 1,000 subjects. A total of 38 different prevalence data points from 31 studies were analyzed using a random-effects, due to high heterogeneity; resulting in a pooled prevalence of 32 (95% confidence interval [CI] 26–38; I^2^: 98.8%) per 1,000 subjects. See Supplementary Figure I.

In the stratified analysis, the pooled prevalence of stroke was similar in men (21; 95% CI 17–25, I^2^: 94.6%) and women (20; 95% CI 16–23, I^2^: 97%).

**Figure 4:**
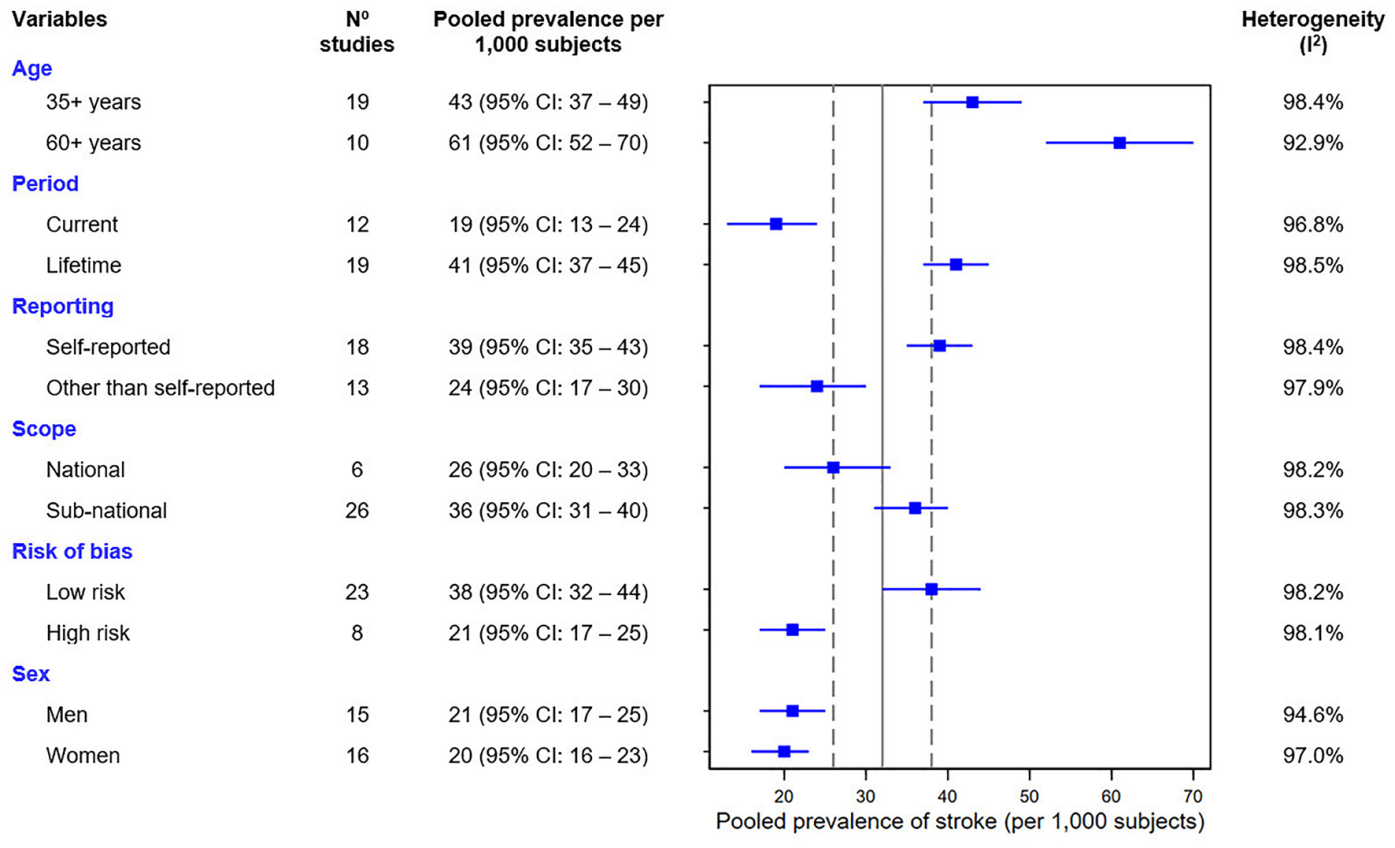
Forest plot of prevalence rate about stroke in subgroup analysis according to different variables of interest. Meta-analysis was calculated by fitting a logistic-normal random-effect model without covariates and heterogeneity was assessed with the statistical test I^2^. Vertical continuous line represents the overall estimate of the prevalence of stroke in the general population and dashed lines represent the 95% confidence interval (CI). Detailed forest plot of the general population prevalence rate in Supplementary Figure I.

In the subgroup analysis, the pooled stroke prevalence of stroke increased from 43 (95% CI 37–49) among studies including individuals aged ≥ 35 years compared to those including subjects ≥ 60 years (61, 95% CI 52–70). The pooled stroke prevalence of stroke was higher among in studies using a lifetime estimate compared to those using a current estimate (41 vs. 19 per 1000 subjects, respectively). Studies with a low risk of bias had a higher pooled stroke prevalence compared to those with a high risk of bias (38 vs. 21 per 1,000 subjects). See details in Fig. 4.

In the meta-regression analysis, studies carried out in the Brazil (*p* = 0.04), Cuba (*p* = 0.03), and Dominican Republic (*p* = 0.01) yielded higher. It was also explained by age (*p* = 0.01), the prevalence increases by 1.1 per 1000 subjects for each additional year in the mean age of the population studied. The method used to report the diagnosis used was also associated with heterogeneity (*p* = 0.04).

### Stroke incidence: pooled estimates

A total of 15 different incidence data points from 11 studies were included in the meta-analysis, with an incidence ranging from 131 to 2970 per 100,000 person-years, a random-effects model was used due to high heterogeneity resulting in a pooled incidence of 255 (95% CI 217–293; I^2^: 99.3%). See Supplementary Figure II.

In the stratified analysis, the pooled incidence among individuals aged ≥ 35 years was 209 (95% CI 163–255) per 100,000 person-years. By according to sex, the pooled incidence of stroke was higher in men (261; 95% CI 222–301) compared to women (218; 95% CI 185–251).

**Figure 5:**
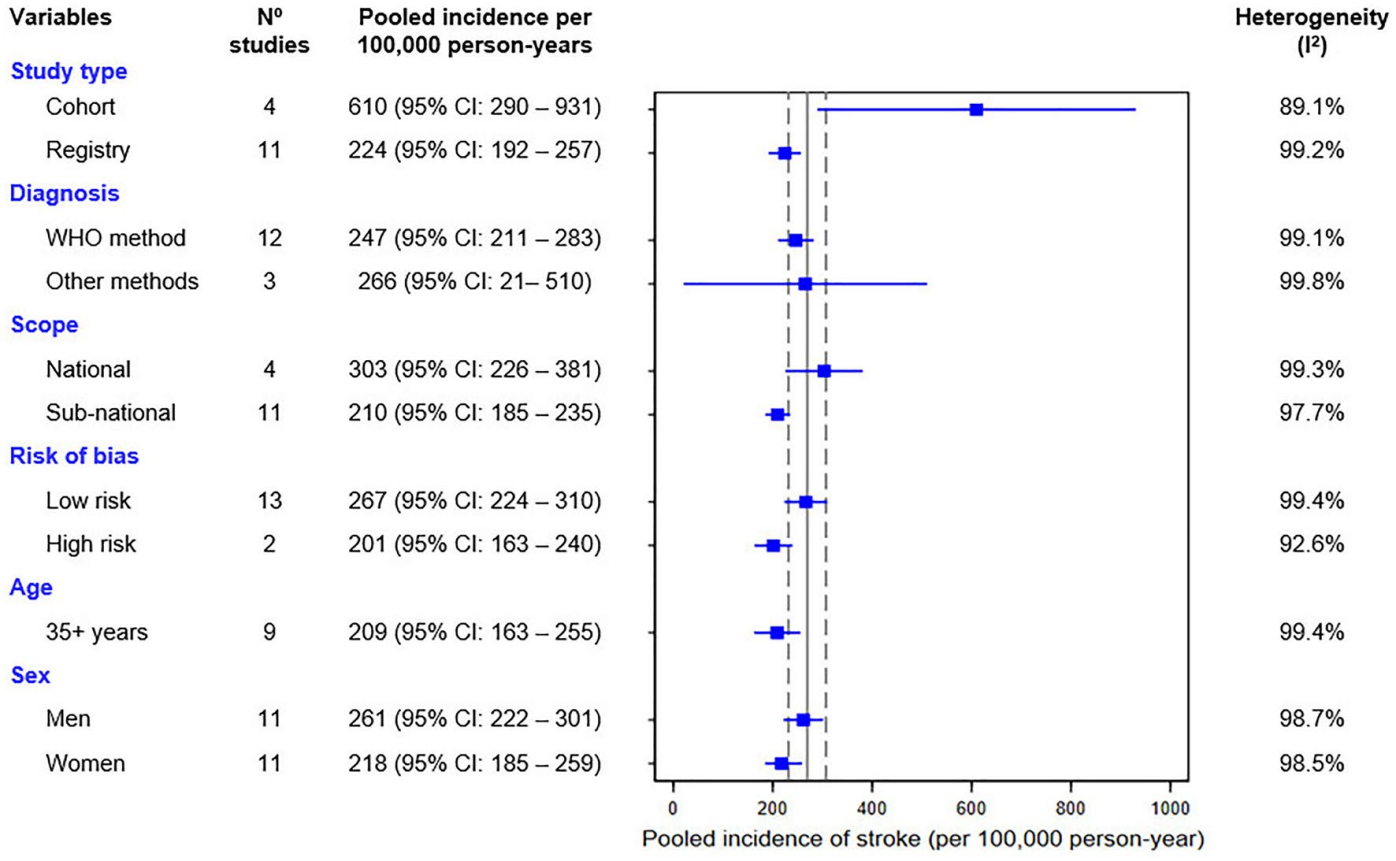
Forest plot of incidence rate about stroke in subgroup analysis according to different variables of interest. Meta-analysis was calculated by fitting a logistic-normal random-effect model without covariates and heterogeneity was assessed with the statistical test I2. Vertical continuous line represents the overall estimate of the incidence of stroke in the general population and dashed lines represent the 95% confidence interval (CI). Detailed forest plot of the general population incidence rate in Supplementary Figure II.

In the subgroup analysis, the pooled estimate of the incidence of stroke was higher among cohort studies compared to those of registries (610 vs. 224 per 100,000 person-years, respectively). Studies using the WHO method to diagnose stroke had a lower pooled incidence compared to studies using other methods such as computerized tomography or hospital cases/surveillance (247 vs. 265 per 100,000 person-years). Studies with a low risk of bias had a higher pooled incidence of stroke compared to those with a high risk of bias (267 vs. 201 per 100,000 person-years). See details in Fig. 5.

In the meta-regression analysis, studies carried out in Cuba and Ecuador yielded higher incidence estimates (*p* = 0.04 and *p* = 0.006, respectively).

## Discussion

In the present meta-analysis, we reviewed the prevalence and incidence of stroke in the LAC region. We re-estimated the prevalence and incidence of stroke in individuals aged 18 and older, due to the high variability in age groups included in the review. Stratified analysis showed similar estimates of stroke prevalence when compared by sex, but higher incidence rates in men than women. Regarding the high heterogeneity, the results varied according to the methodological characteristics of the study as well as the demographic characteristics of the population, reinforcing the need to use appropriate and standardized approaches to obtain adequate population estimates in a region with a high burden of cardiovascular events. Despite the high heterogeneity, our results highlight the relevance of the prevalence and incidence of stroke in LAC countries.

Our pooled prevalence estimate was similar to that of different developed countries. For example, the prevalence of stroke in the adult population in the United States was roughly 3%^60^, whereas this estimate in southwestern China was 3.1%^61^, and 2.9% in Germany^62^. These findings differ from those of a previous systematic review performed in South America^6^ which reported a lower prevalence that that of developed countries. This might be due to the demographic and economic transition with the subsequent rise in risk factors and morbidity related to stroke in most recent studies^4^. Our subgroup analyses showed that older age increase was related to a greater pooled prevalence, with individuals aged ≥ 60 years showing a 41% higher pooled prevalence than subjects ≥ 35 years of age. Although age is a non-modifiable risk factor, it is a factor that plays a relevant role in stroke burden^63^. Prevalence estimates among women and men were similar but slightly higher in men, in contrast to the results of a worldwide systematic review in 2009 in which the pooled prevalence of stroke was higher among men than women (male/female rate ratio: 1.41)^64^; partially explained by a study reported in 2018 that mentions men are more likely to experience a stroke than women because men are more prone to alcoholism and smoking, which are important risk factors for cardiovascular and metabolic diseases^65^. In contrast to the GBD 2019 which described an inverse result with a male/female rate ratio of 0.79^3^. Since there was a difference in the methodology used between the present systematic review and the previous one conducted in 2003^6^, it was not possible to make a further comparison about rates by sex. Meta-regression in prevalence studies shows differences that are statistically significant, so the high heterogeneity across studies (I^2^ = 98.8%) could be attributed to a higher mean age (*p* = 0.01) in the subjects studied, since older adults have a higher range of health conditions and risk factors that can lead to stroke. Another aspect that could be contributing to a high heterogeneity is self-report as a diagnosis method, it biased the pooled prevalence by increasing it. Heterogeneity was explained also by within country variation, especially in the case of Brazil (*p* = 0.04), Cuba (*p* = 0.03) and Dominican Republic (*p* = 0.01). Since most of these studies were not representative at the national level but by community and included populations of different ages, which makes them heterogeneous among themselves.

In the case of incidence studies, our pooled estimate among subjects ≥ 35 years was lower compared to studies in developed countries, such as Spain^66^, or France^67^ with the same population group (220 and 264 per 100,000 person-years, respectively). Men had a 26% higher pooled incidence than women, which is consistent with the aforementioned systematic review in which the pooled incidence was higher in men than in women (male/female rate ratio: 1.33)^64^, but in contrast to the GBD 2019 results describing higher results in women than men (male/female rate ratio 0.89)^3^. The heterogeneity in incidence studies is explained by the variation within the countries, especially in the case of Cuba and Ecuador. These studies were not national in scope, but rather focused on specific communities, demonstrating that these cities are different from the rest.

Although our findings are relevant, limitations must be acknowledged. Results may not be totally representative of the region since Brazil contributed with 35% of the total data points biasing the pooled prevalence and incidence estimates. Self-reporting was a common technique used to estimate stroke prevalence and incidence, which may induce bias due to the number of false-positive cases included in original analyses. Although self-reporting has been validated with a positive predictive value of 79%^68^, it can range from 22 to 87%^69^. To manage this, we analyzed information by risk of bias.

For adequate knowledge of the burden of stroke, we should have included information on stroke subtypes since each subtype may have different risk factors, different initial care in the acute phase and different fatality rates. For example, hemorrhagic stroke is less common than ischemic stroke^70^, and having different clinical presentations may affect diagnostic accuracy. Unfortunately, this could not be obtained as most of the studies included in the review did not provide separate data on stroke subtypes. Given that our review also includes the rural population it is important to acknowledge that this can also be an influence factor in the results. For instance, rural populations usually have less access to healthcare and this might result in underdiagnosis of stroke therefore an under-reporting of the prevalence and incidence of this disease^71^. Over time this can influence the data by leading to biased epidemiology information**.**

The current challenges related to stroke are focused on epidemiological surveillance, health promotion and disease prevention, as well as acute care and rehabilitation. It is imperative to improve the epidemiological surveillance of stroke in different countries of LAC by implementing a good capture system of existing cases, using clinical registries such as electronic health records, standardizing techniques to screen and detecting cases in population-based studies, as recommended by the WHO. We have to take in consideration that public health in the LAC region has many limitations as the access to stroke prevention and rehabilitation, most hospitals are not prepared for stroke care, few healthcare professionals are trained and available to assist acute stroke, few professional training and public awareness campaigns^72^; funding for stroke programs, research, and education is scarce; and national stroke policies are established only in two countries (Brazil and Chile)^73^.

Because the present study focused on outcomes like prevalence and incidence, we could not include some important determinants such as: risk factors, exposure patterns, or ischemic stroke subtypes. The importance of future studies focusing on these determinants is that it could provide a more in-depth approach, which could take closer to the probable cause of the epidemiological characteristics in the LAC region. Studies that can help construct solid estimates of the prevalence and incidence of stroke in LAC are needed. They must allow comparison but also the pooling of information with the use of similar definitions, methods, data analysis and presentation^5^. The WHO MONICA (Monitoring Trends and Determinants in Cardiovascular Disease) Study, which was designed to monitor trends in the incidence of myocardial infarction and stroke is a good example, but the LAC region was not included in this study^74^. The GBD 2019 has been used to obtain estimates of stroke by mathematical modeling instead of using real data, which may not reflect the local epidemiological scenario in LAC countries.

Countries with existing data collection systems can integrate different elements of the WHO Stroke Surveillance System at relatively low costs. This includes an ongoing systematic collection, analysis, interpretation, and dissemination of health information^75^. A surveillance system worthy of imitation would be JOINVASC, a population-based registry in the city of Joinville, Brazil, one of the largest stroke databases in the world. JOINVASC was initiated in 1995 and by 2013 had achieved a reduction of 37% in age-adjusted stroke incidence by obtaining information using the WHO methodology^76^.

A large proportion of the population are not aware that they have risk factors that can lead to stroke. Metabolic risk factors are the main contributors to the overall stroke burden according to an article published in 2022 that mentions that these are mainly high blood pressure and elevated body mass index^77^. Smoking, excessive alcohol consumption, unhealthy diet and sedentary lifestyle have increased in the last years in LAC region specifically in Cuba in comparison to other places such as Europe contributing to the principal risk factors mentioned and therefore increasing incidence of stroke^78^. Mortality and morbidity from cardiometabolic diseases, such as stroke, were associated with suboptimal diet^79^. In comparison to other countries the main diet-related cardiometabolic burdens in LAC were from low intake of nuts and fruits and a high intake of processed meat, which causes toxic metabolic products of the intestinal microbiome and in animal models has been shown to cause atherosclerosis consequently increasing the risk of stroke^80^. The lack of region-specific data about hypertension, metabolic diseases, and atrial fibrillation is also relevant. Identification of these manageable risk factors would have an impact on the long-term appearance of new cases. Preventive interventions should also be encouraged: nonpharmacological and pharmacological (such as aspirin and inexpensive blood pressure-lowering agents)^81^. Finally, we consider the inclusion of manuscripts without language restrictions to be a strength. By not limiting our sources to a single language, we were able to find manuscripts in three different languages, which allowed us to cover a wider range of literature.

In conclusion, our results highlight the relevance of stroke in the LAC region, and the problem seems to be higher among men. LAC countries should develop strong primary prevention and rehabilitation services to provide optimal care for stroke cases.

## Supplementary Information

Supplementary Information.

## Data Availability

The data that support the findings of this study are available from the corresponding author, [DC-C], upon reasonable request.
