# Supplementary material for: Prevalence and incidence of stroke in Latin America and the Caribbean: a systematic review and meta-analysis
Source: Sci Rep. 2023 Apr 26;13:6809. doi: 10.1038/s41598-023-33182-3 (PMC10133252; doi:10.1038/s41598-023-33182-3)
Supplement: Supplementary file 1 — Supplementary Information. [file 41598_2023_33182_MOESM1_ESM.docx]

**SUPPLEMENTAL MATERIAL**



**PREVALENCE AND INCIDENCE OF STROKE IN LATIN AMERICA AND THE CARIBBEAN: A SYSTEMATIC REVIEW AND META-ANALYSIS**

Diego Cagna-Castillo*, MD^1^; A. Lucia Salcedo-Carrillo, MD^1^; Rodrigo M. Carrillo-Larco, MD^2,3^; Antonio Bernabé-Ortiz, MD, MPH, PhD^1,2^

1. Universidad Cientifica del Sur, Lima, Peru
2. CRONICAS Center of Excellence in Chronic Diseases, Universidad Peruana Cayetano Heredia, Lima, Peru
3. Department of Epidemiology and Biostatistics, School of Public Health, Imperial College London, London, UK

**Keywords:** Stroke, Epidemiology, Systematic Review, Prevalence, Incidence.

**Corresponding author:**

Diego Cagna-Castillo, MD

Universidad Científica del Sur, Lima, Peru

Postal address: 15022

Email: [100015338@cientifica.edu.pe](mailto:100015338@cientifica.edu.pe), [diegocagna08@hotmail.com](mailto:diegocagna08@hotmail.com)

Phone: (511) 975-058-787

**INDEX**

[PRISMA 2020 Checklist 3](#_Toc127551508)

[Supplementary Table I. Literature Search Strategy 6](#_Toc127551509)

[Supplementary table I.1 Ovid (Embase, Medline and Global Health) 6](#_Toc127551510)

[Supplementary table I.2 LILACS 6](#_Toc127551511)

[Supplementary table II. Agreement among authors on full-text articles evaluated for eligibility. 7](#_Toc127551512)

[Supplemental Table III. Prevalence studies characteristics 8](#_Toc127551513)

[Supplemental Table IV. Incidence studies characteristics 10](#_Toc127551514)

[Supplemental Figure II. Forest plot and pooled incidence of stroke in the general population. Meta-analysis was calculated by fitting a logistic-normal random-effect model without covariates and heterogeneity was assessed with the statistical test I2. 12](#_Toc127551515)

[References 12](#_Toc127551516)

# PRISMA 2020 Checklist

| **Section and Topic** | **Item #** | **Checklist item** | **Location where item is reported** |
| --- | --- | --- | --- |
| **TITLE** | | |  |
| Title | 1 | Identify the report as a systematic review. | 1 |
| **ABSTRACT** | | |  |
| Abstract | 2 | See the PRISMA 2020 for Abstracts checklist. | 2 |
| **INTRODUCTION** | | |  |
| Rationale | 3 | Describe the rationale for the review in the context of existing knowledge. | 3 |
| Objectives | 4 | Provide an explicit statement of the objective(s) or question(s) the review addresses. | 3 |
| **METHODS** | | |  |
| Eligibility criteria | 5 | Specify the inclusion and exclusion criteria for the review and how studies were grouped for the syntheses. | 3 |
| Information sources | 6 | Specify all databases, registers, websites, organisations, reference lists and other sources searched or consulted to identify studies. Specify the date when each source was last searched or consulted. | 3 |
| Search strategy | 7 | Present the full search strategies for all databases, registers and websites, including any filters and limits used. | Supplemental Material page 6 |
| Selection process | 8 | Specify the methods used to decide whether a study met the inclusion criteria of the review, including how many reviewers screened each record and each report retrieved, whether they worked independently, and if applicable, details of automation tools used in the process. | 3 |
| Data collection process | 9 | Specify the methods used to collect data from reports, including how many reviewers collected data from each report, whether they worked independently, any processes for obtaining or confirming data from study investigators, and if applicable, details of automation tools used in the process. | 4 |
| Data items | 10a | List and define all outcomes for which data were sought. Specify whether all results that were compatible with each outcome domain in each study were sought (e.g. for all measures, time points, analyses), and if not, the methods used to decide which results to collect. | NA |
|  | 10b | List and define all other variables for which data were sought (e.g. participant and intervention characteristics, funding sources). Describe any assumptions made about any missing or unclear information. | NA |
| Study risk of bias assessment | 11 | Specify the methods used to assess risk of bias in the included studies, including details of the tool(s) used, how many reviewers assessed each study and whether they worked independently, and if applicable, details of automation tools used in the process. | 4 |
| Effect measures | 12 | Specify for each outcome the effect measure(s) (e.g. risk ratio, mean difference) used in the synthesis or presentation of results. | 4 |
| Synthesis methods | 13a | Describe the processes used to decide which studies were eligible for each synthesis (e.g. tabulating the study intervention characteristics and comparing against the planned groups for each synthesis (item #5)). | NA |
|  | 13b | Describe any methods required to prepare the data for presentation or synthesis, such as handling of missing summary statistics, or data conversions. | 4 |
|  | 13c | Describe any methods used to tabulate or visually display results of individual studies and syntheses. | 4 |
|  | 13d | Describe any methods used to synthesize results and provide a rationale for the choice(s). If meta-analysis was performed, describe the model(s), method(s) to identify the presence and extent of statistical heterogeneity, and software package(s) used. | 4 |
|  | 13e | Describe any methods used to explore possible causes of heterogeneity among study results (e.g. subgroup analysis, meta-regression). | 4 |
|  | 13f | Describe any sensitivity analyses conducted to assess robustness of the synthesized results. | NA |
| Reporting bias assessment | 14 | Describe any methods used to assess risk of bias due to missing results in a synthesis (arising from reporting biases). | 4 |
| Certainty assessment | 15 | Describe any methods used to assess certainty (or confidence) in the body of evidence for an outcome. | NA |
| **RESULTS** | | |  |
| Study selection | 16a | Describe the results of the search and selection process, from the number of records identified in the search to the number of studies included in the review, ideally using a flow diagram. | 4 |
|  | 16b | Cite studies that might appear to meet the inclusion criteria, but which were excluded, and explain why they were excluded. | NA |
| Study characteristics | 17 | Cite each included study and present its characteristics. | Supplemental Material page 7-9 |
| Risk of bias in studies | 18 | Present assessments of risk of bias for each included study. | 16 |
| Results of individual studies | 19 | For all outcomes, present, for each study: (a) summary statistics for each group (where appropriate) and (b) an effect estimate and its precision (e.g. confidence/credible interval), ideally using structured tables or plots. | 17-18 |
| Results of syntheses | 20a | For each synthesis, briefly summarise the characteristics and risk of bias among contributing studies. | 17-18 |
|  | 20b | Present results of all statistical syntheses conducted. If meta-analysis was done, present for each the summary estimate and its precision (e.g. confidence/credible interval) and measures of statistical heterogeneity. If comparing groups, describe the direction of the effect. | Supplemental Material page 10-11 |
|  | 20c | Present results of all investigations of possible causes of heterogeneity among study results. | 17-18 |
|  | 20d | Present results of all sensitivity analyses conducted to assess the robustness of the synthesized results. | NA |
| Reporting biases | 21 | Present assessments of risk of bias due to missing results (arising from reporting biases) for each synthesis assessed. | NA |
| Certainty of evidence | 22 | Present assessments of certainty (or confidence) in the body of evidence for each outcome assessed. | 17-18 |
| **DISCUSSION** | | |  |
| Discussion | 23a | Provide a general interpretation of the results in the context of other evidence. | 6-7 |
|  | 23b | Discuss any limitations of the evidence included in the review. | 6 |
|  | 23c | Discuss any limitations of the review processes used. | 6 |
|  | 23d | Discuss implications of the results for practice, policy, and future research. | 7 |
| **OTHER INFORMATION** | | |  |
| Registration and protocol | 24a | Provide registration information for the review, including register name and registration number, or state that the review was not registered. | 3 |
|  | 24b | Indicate where the review protocol can be accessed, or state that a protocol was not prepared. | NA |
|  | 24c | Describe and explain any amendments to information provided at registration or in the protocol. | NA |
| Support | 25 | Describe sources of financial or non-financial support for the review, and the role of the funders or sponsors in the review. | 7 |
| Competing interests | 26 | Declare any competing interests of review authors. | 8 |
| Availability of data, code and other materials | 27 | Report which of the following are publicly available and where they can be found: template data collection forms; data extracted from included studies; data used for all analyses; analytic code; any other materials used in the review. | NA |

# Supplementary Table I. Literature Search Strategy

## Supplementary table I.1 Ovid (Embase, Medline and Global Health)

| 1 | Exp stroke/ |
| --- | --- |
| 2 | stroke, lacunar/ |
| 3 | stroke.mp. |
| 4 | stroke disease.mp |
| 5 | cerebrovascular accident.mp |
| 6 | cerebrovascular disease.mp |
| 7 | CVA.mp |
| 8 | cerebral infarction.mp |
| 9 | cerebral ischemia.mp |
| 10 | cerebral hemorrhage.mp |
| 11 | brain infarction.mp |
| 12 | brain stem infarction.mp |
| 13 | apoplexy.mp |
| 14 | brain vascular accident.mp |
| 15 | transient ischemic attack.mp |
| 16 | 1 or 2 or 3 or 4 or 5 or 6 or 7 or 8 or 9 or 10 or 11 or 12 or 13 or 14 or 15 |
| 17 | (("Antigua and Barbuda") or ("Argentina") or ("Bahamas") or ("Barbados") or ("Belize") or ("Bolivia") or ("Brazil") or ("United States Virgin Islands") or ("British Virgin Islands") or ("Chile") or ("Colombia") or ("Costa Rica") or ("Cuba") or ("Dominica") or ("Dominican Republic") or ("Ecuador") or ("El Salvador") or ("Grenada") or ("Guatemala") or ("Guyana") or ("Haiti") or ("Honduras") or ("Jamaica") or ("Mexico") or ("Nicaragua") or ("Panama") or ("Paraguay") or ("Peru") or ("Puerto Rico") or ("Saint Kitts and Nevis") or ("Saint Lucia") or ("Saint Vincent and the Grenadines") or ("Suriname") or ("Trinidad and Tobago") or ("West Indies") or ("Uruguay") or ("Venezuela") or ("Latin America") or latin amer$ or ("South America") or south amer$ or ("Central America") or central amer$ or ("Caribbean Region")) |
| 18 | 16 and 17 |
| 19 | exp animals/ not humans.sh. |
| 20 | 18 not 19 |
| 21 | remove duplicates from 20 |

## Supplementary table I.2 LILACS

| ((accidente cerebrovascular) OR (enfermedad cerebrovascular) OR (derrame cerebral) OR (hemorragia cerebral) OR (infarto cerebral) OR (ataque isquémico transitorio) OR (infarto de tronco cerebral) OR (ACV) OR (apoplejía)) AND (("Antigua y Barbuda") or ("Argentina") or ("Aruba") or ("Bahamas") or ("Barbados") or ("Belice") or ("Bolivia") or ("Brasil") or ("Islas Vírgenes de los Estados Unidos") or ("Islas Vírgenes Británicas") or ("Islas Caimán") or ("Chile") or ("Colombia") or ("Costa Rica") or ("Cuba") or ("Curazao") or ("Dominica") or ("Republica Dominicana") or ("Ecuador") or ("El Salvador") or ("Granada") or ("Guatemala") or ("Guyana") or ("Haití") or ("Honduras") or ("Jamaica") or ("México") or ("Nicaragua") or ("Panamá") or ("Paraguay") or ("Perú") or ("Puerto Rico") or ("San Cristóbal y Nieves ") or ("Santa Lucía") or ("San Vicente y las Granadinas ") or ("Surinam") or ("Trinidad y Tobago") or ("Turcas y Caicos ") or ("Uruguay") or ("Venezuela") or ("América Latina") or ("Latinoamérica") or ("América del Sur") or ("Sudamérica") or ("Suramérica​") or ("América Central") or ("Centroamérica") or ("América del Centro") or ("Caribe")) |
| --- |

# Supplementary table II. Agreement among authors on full-text articles evaluated for eligibility.

|  |  | Author 2 (ALS-C) | | Total |
| --- | --- | --- | --- | --- |
|  |  | Inclusion | Exclusion |  |
| Author 1 (DC-C) | Inclusion | 67 | 3 | 70 |
|  | Exclusion | 7 | 16 | 23 |
|  | Total | 74 | 18 |  |
| Percentage of agreement: 89.24% | | | | |
| Cohen's kappa coefficient: 0.693 | | | **Substantial agreement** | |

# Supplemental Table III. Prevalence studies characteristics

| First author, year | Country | City | Population | Scope | Design | Year of data collection | Age range | Age mean | % Women | Effective sample size | Diagnosis method | Type of stroke classification | Prevalence type |
| --- | --- | --- | --- | --- | --- | --- | --- | --- | --- | --- | --- | --- | --- |
| Gracia, 1988 ^1^ | Panama | Belisairo Porras (San Miguelito) | Urban / rural | Community | Cross-sectional | 1986 | 0+ (25+*) | NR | 43.3 | 955 | WHO | General | Current |
| Nicoletti, 2000 ^2^ | Bolivia | Cordillera (Santa Cruz) | Rural | Community | Cross-sectional | 1994-1996 | 0+ (35+*) | NR | 49.4 | 9,955 | WHO | Hemorrhagic and Ischemic | Current |
| Pradilla, 2002 ^3^ | Colombia | Aratoca (Santander) | Rural | Community | Cross-sectional | 2001 | 0+ (40+*) | NR | 60.3 | 1,586 | WHO | General | Current |
| Pradilla, 2003 ^4^ | Colombia | Bogota (Bogota DC), Cali (Valle del Cauca) Medellin (Antioquia), Bucaramanga (Santander), Barranquilla (Atlántico) | Urban / rural | National | Cross-sectional | 1995-1996 | 0+ (50+*) | NR | 60.0 | 8,910 | WHO | General | Current |
| Patel, 2006 ^5^ | Mexico |  |  | National | Cross-sectional | 2001 | 65+ | 73.0 | 52.4 | 4,811 | Self-reported | General | Lifetime |
| Diaz-Cabezas, 2006 ^6^ | Colombia | Caldas | Urban / rural | Community | Cross-sectional | 2004-2005 | 7+ (20+*) | 35.4 | 68.0 | 787 | WHO modified | General | Current |
| Melcon, 2006 ^7^ | Argentina | Junin (Buenos Aires) | Rural | Community | Cross-sectional | 1991-1992 | 0+ (40+*) | NR | 53.2 | 17,049 | WHO | General | Current |
| Thompson, 2007 ^8^ | Honduras | Salama (Olancho) | Rural | Community | Cross-sectional | NR | 0+ (25+*) | NR | 52.3 | 5,608 | WHO | General | Current |
| Giacomin, 2008 ^9^ | Brazil | Minas Gerais (Belo Horizonte) |  | Community | Cross-sectional | 2003 | 60+ | 69.7 | 58.9 | 1,786 | Self-reported | General | Lifetime |
| Kuri-Morales, 2009 ^10^ | Mexico | Mexico City | Urban | Community | Cross-sectional | 1998-2004 | 35-84 | NR | 67.0 | 157,081 | Self-reported | General | Lifetime |
| Lima, 2009 ^11^ | Brazil | Sao Paulo | Urban | Community | Cross-sectional | 2001-2002 | 60+ | 69.9 | 57.2 | 1,958 | Self-reported | General | Lifetime |
| Pereira, 2009 ^12^ | Brazil | Vassouras (Rio de Janeiro) | Urban / rural | Community | Cross-sectional | 2007 | 0+ (60+*) | NR | 54.9 | 4,154 | WHO | Hemorrhagic and Ischemic | Current |
| Sousa, 2009 ^13^ | Cuba | Havana City and Matanzas | Urban / rural | Community | Cross-sectional | 2003-2005 | 65+ | 75.1 | 65.0 | 2,937 | Self-reported | General | Lifetime |
| Sousa, 2009 ^13^ | Dominican Republic |  |  | Community | Cross-sectional | 2003-2005 | 65+ | 75.2 | 66.0 | 2,001 | Self-reported | General | Lifetime |
| Sousa, 2009 ^13^ | Peru |  |  | Community | Cross-sectional | 2003-2005 | 65+ | 74.8 | 60.9 | 1,932 | Self-reported | General | Lifetime |
| Sousa, 2009 ^13^ | Venezuela |  |  | Community | Cross-sectional | 2003-2005 | 65+ | 72.5 | 63.0 | 1,947 | Self-reported | General | Lifetime |
| Sousa, 2009 ^13^ | Mexico |  |  | Community | Cross-sectional | 2003-2005 | 65+ | 74.3 | 63.0 | 2,002 | Self-reported | General | Lifetime |
| Fillenbaun, 2010 ^14^ | Brazil | Rio Grande do Sul | Urban / rural | Subnational | Cross-sectional | 1995 | 60+ | NR | 66.0 | 6,958 | Self-reported | General | Lifetime |
| Cantu-Brito, 2011 ^15^ | Mexico | Durango | Urban / rural | Subnational | Cross-sectional | 2008-2009 | 35+ | 51.5 | 56.7 | 2,437 | WHO | General | Current |
| Andrade, 2012 ^16^ | Brazil | Sao Paulo |  | Community | Cross-sectional | 2000-2001 | 60+ | NR | 58.5 | 2,054 | Self-reported | General | Lifetime |
| Bansilal, 2012 ^17^ | Grenada | St. George |  | National | Cross-sectional | 2008-2009 | 18+ | NR | 58.0 | 2,827 | Self-reported | General | Lifetime |
| Copstein, 2013 ^18^ | Brazil | Porto Alegre |  | Community | Cross-sectional | 2009 | 20+ | 44.1 | 55.9 | 3,391 | 4 item- questionnaire | General | Lifetime |
| Santos Machado, 2013 ^19^ | Brazil | Campinas (Sao Paulo) |  | Community | Cross-sectional | 2011 | 50+ | NR | 100.0 | 622 | Self-reported | General | Lifetime |
| Del Brutto, 2013 ^20^ | Ecuador | Atahualpa (El oro) | Rural | Community | Cross-sectional | 2003 | 40+ | NR | NR | 642 | WHO | General | Current |
| Alexandre, 2014 ^21^ | Brazil | Sao Paulo | Urban | Community | Longitudinal | 2006 | 60+ | 69.6 | 61.8 | 1,413 | Self- reported | General | Lifetime |
| Fernandes, 2014 ^22^ | Brazil | Coari | Urban / rural | Community | Cross-sectional | 2011 | 35+ | 59.2 | 56.8 | 5,925 | WHO | General | Current |
| Gracia, 2014 ^23^ | Panama | Panama city | Urban | National | Cross-sectional | 2007 | NR | 42.6 | 60.1 | 25,748 | Self-reported | General | Lifetime |
| Gracia, 2014 ^23^ | Panama | Panama city | Urban | Community | Cross-sectional | 2010 | NR | 45.4 | 70.1 | 3,590 | Self-reported | General | Lifetime |
| Theme Filha, 2015 ^24^ | Brazil |  |  | National | Cross-sectional | 2013 | 18+ | NR | 52.0 | 60,202 | Self-reported | General | Lifetime |
| Downer, 2016 ^25^ | Mexico |  |  | National | Longitudinal | 2000-2001 | 80+ | 84.7 | 56.8 | 761 | Self-reported | General | Lifetime |
| Downer, 2016 ^25^ | Mexico |  |  | National | Longitudinal | 2010-2012 | 80+ | 84.8 | 56.6 | 1,426 | Self-reported | General | Lifetime |
| Avezum, 2017 ^26^ | Argentina |  |  | Community | Cross-sectional | 2003-2009 | 35-70 | NR | 61.5 | 7,534 | Self-reported | General | Lifetime |
| Avezum, 2017 ^26^ | Brazil |  |  | Community | Cross-sectional | 2003-2009 | 35-70 | NR | 55.1 | 6,081 | Self-reported | General | Lifetime |
| Gondim, 2017 ^27^ | Brazil |  |  | Community | Cross-sectional | NR | 60+ | NR | 72.0 | 418 | Self-reported | General | Lifetime |
| Miranda, 2019 ^28^ | Peru |  |  | Subnational | Cross-sectional | 2010 | 35+ | NR | 50.7 | 2,890 | Self-reported | General | Lifetime |
| Bernardes, 2019 ^29^ | Brazil | Belo Horizonte |  | Community | Cross-sectional | 2010 | 60+ | NR | 61.0 | 2,172 | Self-reported | General | Lifetime |
| Ameriso, 2020 ^30^ | Argentina | General Villegas (Buenos aires) |  | Community | Cross-sectional | 2015-2016 | 40+ | NR | NR | 2,180 | WHO | Hemorrhagic and Ischemic | Current |
| Camacho, 2020 ^31^ | Colombia |  |  | Subnational | Cross-sectional | 2005-2009 | 35-70 | 50.7 | 64.1 | 7,485 | Self-reported | General | Lifetime |

NR: not reported

*: Estimated for the age indicated

# Supplemental Table IV. Incidence studies characteristics

| First author | Country | City | Population | Scope | Design | Period | Age range | Mean age | % Women | Sample size | Diagnosis method | Type of stroke classification |
| --- | --- | --- | --- | --- | --- | --- | --- | --- | --- | --- | --- | --- |
| Corbin, 2004 ^32^ | Barbados |  |  | National | Registry | 2001-2002 | 0+ (25+*) | NR | NR | NR | WHO | General |
| Lavados, 2005 ^33^ | Chile | Iquique |  | Community | Registry | 2000-2002 | 0+ (25+*) | NR | NR | NR | Hospital cases | General |
| Hochmann, 2006 ^34^ | Uruguay | Rivera |  | Community | Registry | 2000-2001 | 35+ | NR | NR | NR | WHO | Hemorrhagic and Ischemic |
| Cantu-Brito, 2011 ^15^ | Mexico | Durango | Urban/rural | Community | Cross-sectional | 2007-2008 | 35+ | 51.5 | 56.7 | 2437 | Hospital surveillance | General |
| Olindo, 2014 ^35^ | Martinique |  |  | National | Registry | 1998-1999 | 0+ (35+*) | NR | NR | NR | WHO | General |
| Olindo, 2014 ^35^ | Martinique |  |  | National | Registry | 2011-2012 | 0+ (35+*) | NR | NR | NR | WHO | General |
| Llibre-Guerra, 2015 ^36^ | Cuba | La Habana, Matanzas |  | Community | Cohort | 2003-2007 | 65+ | NR | 65.3 | 2802 | WHO | General |
| Cabral, 2016 ^37^ | Brazil | Joinville (Santa Catarina) |  | Community | Registry | 1995 | 0+ (25+*) | NR | NR | NR | WHO | General |
| Cabral, 2016 ^37^ | Brazil | Joinville (Santa Catarina) |  | Community | Registry | 2005-2006 | 0+ (25+*) | NR | NR | NR | WHO | General |
| Cabral, 2016 ^37^ | Brazil | Joinville (Santa Catarina) |  | Community | Registry | 2012-2013 | 0+ (25+*) | NR | NR | NR | WHO | General |
| Bahit, 2016 ^38^ | Argentina | Tandil |  | Community | Registry | 2013-2015 | 0+ (25+*) | NR | NR | NR | WHO | General |
| Del Brutto, 2017 ^39^ | Ecuador | Atahualpa | Rural | Community | Cohort | 2012-2016 | 40+ | 57.0 | 56.0 | 807 | WHO | General |
| Meza, 2020 ^40^ | Mexico |  |  | National | Cohort | 2001-2015 | 50+ | 62.0 | 54.8 | 10693 | Self-report | General |
| Minelli, 2020 ^41^ | Brazil | Matão (São Paulo) | Urban | Community | Registry | 2003-2004 | 0+ (45+) | NR | NR | NR | WHO | General |
| Minelli, 2020 ^41^ | Brazil | Matão (São Paulo) | Urban | Community | Registry | 2015-2016 | 0+ (45+*) | NR | NR | NR | WHO | General |

NR: not reported

* Estimated for the age indicated

**Supplemental Figure I. Forest plot and pooled prevalence of stroke in the general population.** Meta-analysis was calculated by fitting a logistic-normal random-effect model without covariates and heterogeneity was assessed with the statistical test I2.


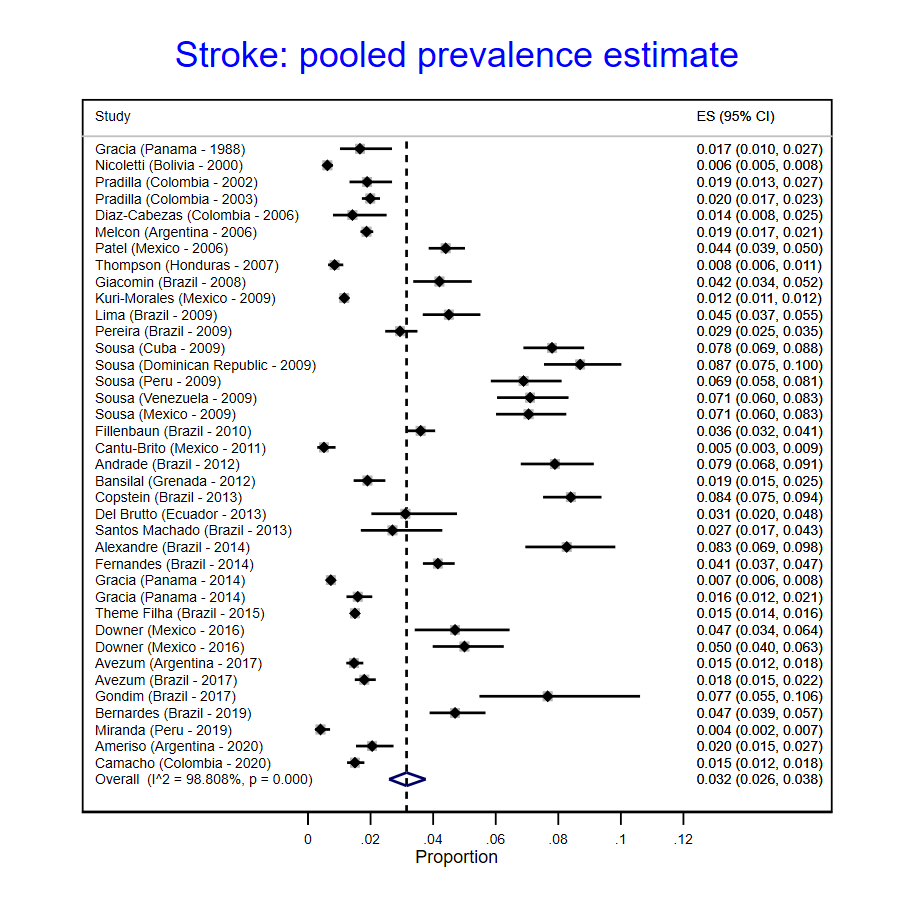


# Supplemental Figure II. Forest plot and pooled incidence of stroke in the general population. Meta-analysis was calculated by fitting a logistic-normal random-effect model without covariates and heterogeneity was assessed with the statistical test I2.


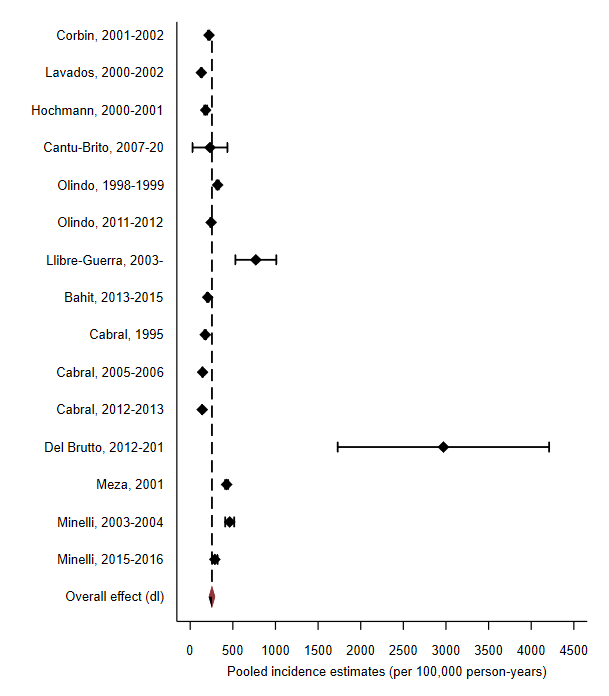


# References

1. Gracia FJ, Bayard V, Triana E, et al. Prevalencia de enfermedades neurologica en el corregimiento de Belisario Porras, Distrito di San Miguelito, en Panama, 1986. *Rev Méd Panamá* 1988; 13: 40–45.

2. Nicoletti A, Sofia V, Giuffrida S, et al. Prevalence of Stroke: A Door-to-Door Survey in Rural Bolivia. *Stroke* 2000; 31: 882–885.

3. Pradilla Ardila G, Vesga Angarita BE, León-Sarmiento FE. Estudio neuroepidemiológico en Aratoca, una área rural del oriente colombiano. *Rev Médica Chile*; 130. Epub ahead of print February 2002. DOI: 10.4067/S0034-98872002000200009.

4. Pradilla A. G, Vesga A. BE, León-Sarmiento FE. Estudio neuroepidemiológico nacional (EPINEURO) colombiano. *Rev Panam Salud Pública* 2003; 14: 104–111.

5. Patel KV, Peek MK, Wong R, et al. Comorbidity and Disability in Elderly Mexican and Mexican American Adults: Findings From Mexico and the Southwestern United States. *J Aging Health* 2006; 18: 315–329.

6. Díaz Cabezas R, Ruano Restrepo MI, Chacón Cardona JA, et al. Perfil neuroepidemiológico en la zona centro del departamento de Caldas (Colombia), años 2004-2005. *Rev Neurol* 2006; 43: 646.

7. Melcon CM, Melcon MO. Prevalence of Stroke in an Argentine Community. *Neuroepidemiology* 2006; 27: 81–88.

8. Thompson Cerna A, Medina Hernández MT. Prevalencia de la enfermedad cerebrovascular en la comunidad rural de Salamá, Honduras, utilizando el método epidemiológico de captura-recaptura. *Rev Neurol* 2007; 44: 460.

9. Giacomin KC, Peixoto SV, Uchoa E, et al. Estudo de base populacional dos fatores associados à incapacidade funcional entre idosos na Região Metropolitana de Belo Horizonte, Minas Gerais, Brasil. *Cad Saúde Pública* 2008; 24: 1260–1270.

10. Kuri-Morales P, Emberson J, Alegre-Díaz J, et al. The prevalence of chronic diseases and major disease risk factors at different ages among 150 000 men and women living in Mexico City: cross-sectional analyses of a prospective study. *BMC Public Health* 2009; 9: 9.

11. Lima MG, Barros MB de A, César CLG, et al. Impact of chronic disease on quality of life among the elderly in the state of São Paulo, Brazil: a population-based study. *Rev Panam Salud Pública*; 25. Epub ahead of print April 2009. DOI: 10.1590/S1020-49892009000400005.

12. Pereira ABCN da G, Alvarenga H, Pereira Júnior RS, et al. Prevalência de acidente vascular cerebral em idosos no Município de Vassouras, Rio de Janeiro, Brasil, através do rastreamento de dados do Programa Saúde da Família. *Cad Saúde Pública* 2009; 25: 1929–1936.

13. Sousa RM, Ferri CP, Acosta D, et al. Contribution of chronic diseases to disability in elderly people in countries with low and middle incomes: a 10/66 Dementia Research Group population-based survey. *The Lancet* 2009; 374: 1821–1830.

14. Fillenbaum GG, Blay SL, Andreoli SB, et al. Prevalence and Correlates of Functional Status in an Older Community— Representative Sample in Brazil. *J Aging Health* 2010; 22: 362–383.

15. Cantu-Brito C, Majersik JJ, Sánchez BN, et al. Door-to-Door Capture of Incident and Prevalent Stroke Cases in Durango, Mexico: The Brain Attack Surveillance in Durango Study. *Stroke* 2011; 42: 601–606.

16. Andrade FCD, Guevara PE, Lebrão ML, et al. Correlates of the incidence of disability and mortality among older adult Brazilians with and without diabetes mellitus and stroke. *BMC Public Health* 2012; 12: 361.

17. Bansilal S, Vedanthan R, Woodward M, et al. Cardiovascular Risk Surveillance to Develop a Nationwide Health Promotion Strategy: The Grenada Heart Project. *Glob Heart* 2012; 7: 87.

18. Copstein L, Fernandes JG, Bastos GAN. Prevalence and risk factors for stroke in a population of Southern Brazil. *Arq Neuropsiquiatr* 2013; 71: 294–300.

19. de S. Santos Machado V, Valadares ALR, Costa-Paiva LH, et al. Aging, obesity, and multimorbidity in women 50 years or older: a population-based study. *Menopause* 2013; 20: 818–824.

20. Lama J, Del Brutto OH. The Importance of Neurocysticercosis in Stroke in Rural Areas of a Developing Latin American Country. *Am J Trop Med Hyg* 2013; 89: 374–375.

21. Alexandre T da S, Corona LP, Nunes DP, et al. Similarities Among Factors Associated With Components of Frailty in Elderly: SABE Study. *J Aging Health* 2014; 26: 441–457.

22. Fernandes TG, Benseñor IM, Goulart AC, et al. Stroke in the Rain Forest: Prevalence in a  ***Ribeirinha***  Community and an Urban Population in the Brazilian Amazon. *Neuroepidemiology* 2014; 42: 235–242.

23. Gracia F, Benzadon A, Gonzalez-Castellon M, et al. The Impact of Cerebrovascular Disease in Panama. *Int J Stroke* 2014; 9: 28–30.

24. Theme Filha MM, Souza Junior PRB de, Damacena GN, et al. Prevalência de doenças crônicas não transmissíveis e associação com autoavaliação de saúde: Pesquisa Nacional de Saúde, 2013. *Rev Bras Epidemiol* 2015; 18: 83–96.

25. Downer B, Chen N-W, Wong R, et al. Self-Reported Health and Functional Characteristics of Mexican and Mexican American Adults Aged 80 and Over. *J Aging Health* 2016; 28: 1239–1255.

26. Avezum A, Oliveira GBF, Lanas F, et al. Secondary CV Prevention in South America in a Community Setting: The PURE Study. *Glob Heart* 2017; 12: 305.

27. Gondim AS, Coelho Filho JM, Cavalcanti A de A, et al. Prevalence of functional cognitive impairment and associated factors in Brazilian community-dwelling older adults. *Dement Neuropsychol* 2017; 11: 32–39.

28. Miranda JJ, Bernabe-Ortiz A, Gilman RH, et al. Multimorbidity at sea level and high-altitude urban and rural settings: The CRONICAS Cohort Study. *J Comorbidity* 2019; 9: 2235042X1987529.

29. Bernardes GM, Mambrini JV de M, Lima-Costa MF, et al. Perfil de multimorbidade associado à incapacidade entre idosos residentes na Região Metropolitana de Belo Horizonte, Brasil. *Ciênc Saúde Coletiva* 2019; 24: 1853–1864.

30. Ameriso S, Gomez-Schneider M, Hawkes M, et al. Prevalence of stroke in Argentina: A door-to-door population-based study (EstEPA). *Int J Stroke* 2021; 16: 280–287.

31. Camacho PA, Gomez-Arbelaez D, Otero J, et al. Self-Reported Prevalence of Chronic Non-Communicable Diseases in Relation to Socioeconomic and Educational Factors in Colombia: A Community-Based Study in 11 Departments. *Glob Heart* 2020; 15: 35.

32. Corbin DOC, Poddar V, Hennis A, et al. Incidence and Case Fatality Rates of First-Ever Stroke in a Black Caribbean Population: The Barbados Register of Strokes. *Stroke* 2004; 35: 1254–1258.

33. Lavados PM, Sacks C, Prina L, et al. Incidence, 30-day case-fatality rate, and prognosis of stroke in Iquique, Chile: a 2-year community-based prospective study (PISCIS project). *The Lancet* 2005; 365: 2206–2215.

34. Hochmann Piñeiro B, Coelho Santarcieri J, Segura Álvez J, et al. Incidencia del accidente cerebrovascular en la ciudad de Rivera, Uruguay. *Rev Neurol* 2006; 43: 78.

35. Olindo S, Chausson N, Mejdoubi M, et al. Trends in Incidence and Early Outcomes in a Black Afro-Caribbean Population From 1999 to 2012: Etude Réalisée en Martinique et Centrée sur l’Incidence des Accidents Vasculaires Cérébraux II Study. *Stroke* 2014; 45: 3367–3373.

36. Llibre-Guerra JC, Valhuerdi Cepero A, Fernández Concepción O, et al. Incidencia y factores de riesgo de ictus en La Habana y Matanzas, Cuba. *Neurología* 2015; 30: 488–495.

37. Cabral NL, Cougo-Pinto PT, Magalhaes PSC, et al. Trends of Stroke Incidence from 1995 to 2013 in Joinville, Brazil. *Neuroepidemiology* 2016; 46: 273–281.

38. Bahit MC, Coppola ML, Riccio PM, et al. First-Ever Stroke and Transient Ischemic Attack Incidence and 30-Day Case-Fatality Rates in a Population-Based Study in Argentina. *Stroke* 2016; 47: 1640–1642.

39. Del Brutto OH, Mera RM, Zambrano M, et al. Severe edentulism is a major risk factor influencing stroke incidence in rural Ecuador (The Atahualpa Project). *Int J Stroke* 2017; 12: 201–204.

40. Meza E, Eng CW, Sáenz JL, et al. Elevated Depressive Symptoms and the Risk of Stroke among the Mexican Older Population. *J Am Geriatr Soc* 2020; 68: 2579–2586.

41. Minelli C, Cabral NL, Ujikawa LT, et al. Trends in the Incidence and Mortality of Stroke in Matão, Brazil: The Matão Preventing Stroke (MAPS) Study. *Neuroepidemiology* 2020; 54: 75–82.
